# Hydrogen: A Novel Option in Human Disease Treatment

**DOI:** 10.1155/2020/8384742

**Published:** 2020-09-05

**Authors:** Mengling Yang, Yinmiao Dong, Qingnan He, Ping Zhu, Quan Zhuang, Jie Shen, Xueyan Zhang, Mingyi Zhao

**Affiliations:** ^1^Department of Pediatrics, The Third Xiangya Hospital, Central South University, Hunan Province, Changsha 410013, China; ^2^Guangdong Cardiovascular Institute, Guangdong Provincial People's Hospital, Guangdong Academy of Medical Sciences, Guangzhou, Guangdong 510100, China; ^3^Transplantation Center of the 3rd Xiangya Hospital, Central South University, Hunan Province, Changsha 410013, China; ^4^Xiangya School of Medicine, Central South University, Hunan Province, Changsha 410013, China

## Abstract

H_2_ has shown anti-inflammatory and antioxidant ability in many clinical trials, and its application is recommended in the latest Chinese novel coronavirus pneumonia (NCP) treatment guidelines. Clinical experiments have revealed the surprising finding that H_2_ gas may protect the lungs and extrapulmonary organs from pathological stimuli in NCP patients. The potential mechanisms underlying the action of H_2_ gas are not clear. H_2_ gas may regulate the anti-inflammatory and antioxidant activity, mitochondrial energy metabolism, endoplasmic reticulum stress, the immune system, and cell death (apoptosis, autophagy, pyroptosis, ferroptosis, and circadian clock, among others) and has therapeutic potential for many systemic diseases. This paper reviews the basic research and the latest clinical applications of H_2_ gas in multiorgan system diseases to establish strategies for the clinical treatment for various diseases.

## 1. Introduction

Molecular hydrogen (H_2_) is the lightest chemical element in the earth's atmosphere. H_2_ is often mixed in gas cylinders for deep-sea divers to breathe, to prevent decompression and nitrogen sickness [1]. In mammals, H_2_ is spontaneously produced by intestinal bacteria in the process of anaerobic metabolism to produce energy and is enzymatically catabolized by hydrogenases to provide electrons.

Therapeutic applications of H_2_ were first described in 1975. Dole et al. reported that hyperbaric hydrogen caused marked regression of tumors in mice with skin squamous carcinoma [2]. However, hyperbaric H_2_ is not a clinically feasible option, and H_2_ is a physiologically inert gas that seems not to react with any active substances, including oxygen gas, in mammalian cells. Thus, H_2_ was perceived as being nonfunctional and was disregarded clinically.

In 2007, the potential therapeutic benefits of H_2_ were described. Ohsawa et al. discovered that H_2_ has selective antioxidant properties that protect the brain against ischemia/reperfusion (I/R) injury and stroke by specifically neutralizing hydroxyl radicals (^·^OH) and peroxynitrite (ONOO-) but not superoxide anion radical (^·^O_2_-), hydrogen peroxide (H_2_O_2_), and nitric oxide (NO) [3]. The report generated worldwide attention and thrust H_2_ into the spotlight of therapeutic medical gas research. Many studies using cellular, animal, and clinical experiments in a variety of biomedical fields have explored the therapeutic and preventive effects of H_2_. The collective data have indicated that H_2_ is an important pathophysiological regulatory factor with antioxidative, anti-inflammatory, and antiapoptotic effects on cells and organs [4–6]. It is so convenient to use that H_2_ can be easily administered in various ways, including inhalation, injection of H_2_-rich saline (HRS), drinking H_2_-rich water (HW), bathing in HW, and using HRS eyedrops. As well, the production of intestinal H_2_ by bacteria can be increased via oral administration of acarbose and lactulose. Liu and his colleagues demonstrated that the hydrogen concentration reached a peak of 5 min after oral and intraperitoneal administration, and in only 1 min following intravenous administration [7].

Beginning on 31 December 2019 in Wuhan, China, illness and pneumonia named coronavirus disease-2019 (COVID-19) caused by severe acute respiratory syndrome coronavirus 2 (SARS-CoV-2) has spread to become a pandemic. The seventh edition of *Chinese Clinical Guidance for COVID-19 Pneumonia Diagnosis and Treatment (7^th^ edition)* issued by China National Health Commission recommended the inhalation of oxygen mixed with hydrogen gas (33.3% O_2_ and 66.6% H_2_), bringing H_2_ to the forefront of contemporary therapeutic medical gas research.

The preventive and therapeutic effects of H_2_ have been intensively investigated for various pathological processes. In this review, we summarize the most recently published literature concerning the use of H_2_ in respiratory, cardiovascular, nervous, digestive, reproductive, urinary, motor, and sensory system diseases, as well as for the treatment of metabolic syndrome and cancer. We also briefly discuss some known mechanisms underlying the action of H_2_. We hope that this information will increase the understanding of the therapeutic activities of H_2_ and inform future H_2_-based therapies.

## 2. Mechanisms of the Action of H_2_

**Figure 1:**
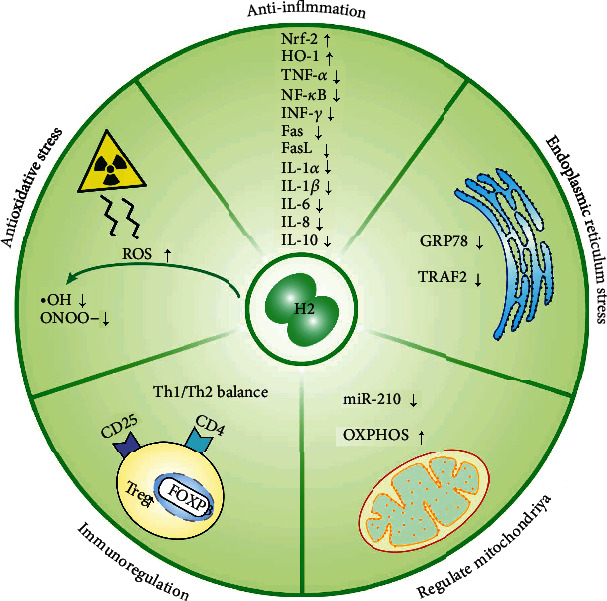
Biological effects of H_2_. H_2_ exhibits selective antioxidative and anti-inflammatory activities and can regulate ER stress, mitochondria, and immune function. H_2_ selectively scavenges ^·^OH and ONOO-, upregulates Nrf-2 and HO-1, and downregulates the expression of proinflammatory and inflammatory cytokines that include TNF-*α*, NF-*κ*B, INF-*γ*, Fas, FasL, IL-1*α*, IL-1*β*, IL-6, IL-8, and IL-10, as well as downregulates ER stress-related factors that include GRP78 and TRAF2. H_2_ also reduces miR-210 and activates OXPHOS in mitochondria. Finally, H_2_ increases CD4+CD25+Foxp3+Treg cells and maintains the Th1/Th2 balance.

**Figure 2:**
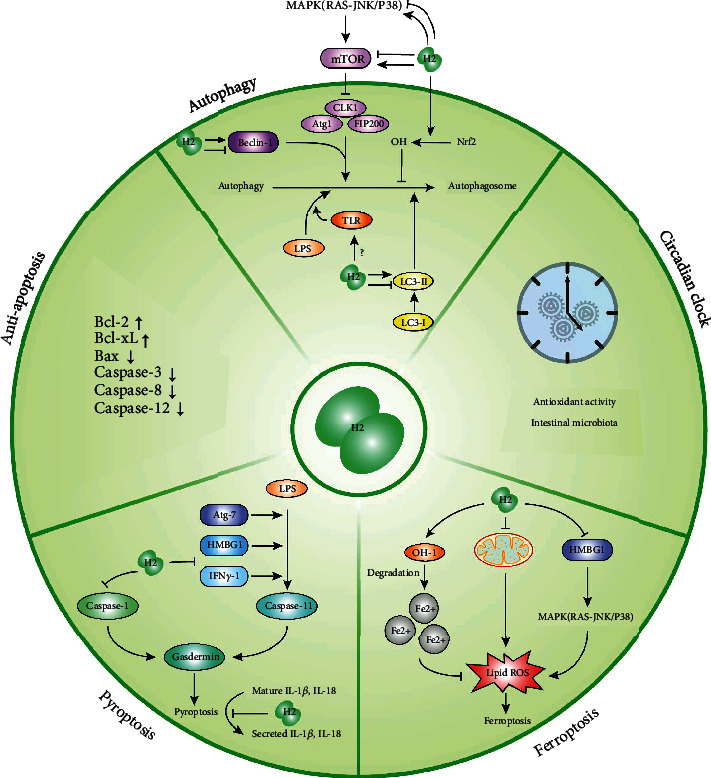
Various effects of H_2_ on cell death. Activities of H_2_ include inhibition of apoptosis, and the regulation of autophagy, circadian clock, ferroptosis, and pyroptosis.

To fully explain the preventive and therapeutic effects of H_2_, biological effects and possible mechanisms are summarized in Figures 1 and 2.

### 2.1. Anti-Inflammatory Effect of H_2_

The anti-inflammatory effect of H_2_ has already been reported in many studies. In the early stage of inflammation, H_2_ can reduce the infiltration of neutrophils and M1 macrophages, and the release of proinflammatory factors by downregulating the expression of intercellular cell adhesion molecule-1 (ICAM-1), granulocyte-macrophage colony-stimulating factor (GM-CSF), and granulocyte colony-stimulating factor (G-CSF) [4]. H_2_ can also inhibit the expression of proinflammatory cytokines during the progress of inflammation and has been revealed in many animal models to decrease the overexpression of early proinflammatory cytokines, such as interleukin- (IL-) 1*β*, IL-6, IL-8, IL-10, tumor necrosis factor-alpha (TNF-*α*) [8], interferon-gamma (INF-*γ*), and late proinflammatory cytokines, such as high-mobility group box-1 protein (HMGB1) [9]. Tanaka and colleagues [10] conducted a gene array analysis of lung grafts from donor rats pretreated with hydrogen ventilation. The authors described that pretreatment with H_2_ obviously elevated the expression of two stress-response proteins: heat shock protein A5 (HSPA5) and dual-specificity phosphatase 1 (DUSP1). HSP70 protein encoded by HSPA5 has anti-inflammatory and antiapoptotic properties. DUSP1 can suppress excessive autophagy by inactivating mitogen-activated protein kinases (MAPKs) to alleviate the inflammatory response. Therefore, we speculate that H_2_ can positively regulate the expression of stress-response proteins and improve the anti-inflammatory ability of the organs. Another study [11] demonstrated ventilation with 2% H_2_ in the air significantly reduced the mRNA levels of the early growth responsive gene-1 (Egr-1) and chemokine (C-C motif) ligand 2 (CCL2), suggesting that H_2_ can affect the progress of inflammation by regulating the transcription of proinflammatory regulatory factors and chemokines. In many diseases, such as airway inflammation and cerebral ischemia, type I hypersensitivity caused by mast cell activation will aggravate the tissue congestion and edema [12, 13]. H_2_ inhibits the body's inflammatory response by inhibiting Th2 reaction to reduce mast cell activation as well [14]. H_2_ can also regulate inflammation by regulating the physiological pathway of T cells. For example, hydrogen treatment can inhibit the overactivation of the immune system by restoring the loss of regulatory T cells (Tregs) [15] and alleviates inflammation by preventing activation-regulated chemokine-mediated T-cell chemotaxis. The regulation effects of H_2_ on programmed cell death, oxidative stress, and mitochondrial function are closely related to the inflammatory response. However, the anti-inflammatory effect of hydrogen cannot be overstated. It would be better to be used as a supplementary therapy.

### 2.2. Regulation of Oxidative Stress

Many studies have established the antioxidant activity of H_2_. However, recently, a study [16] has shown that hydrogen can rapidly and slightly increase 8-hydroxy-2′-deoxyguanosine (8-OHdG) in urine, a marker for oxidative stress, to a similar level as the increase caused by exercise. Exercise-induced generation of reactive oxygen species (ROS) is crucial in cell adaptation, and short-term exposure to low levels of ROS can protect neurons from oxidative stress that would otherwise be lethal [5]. H_2_ may act as a mitohormetic effector to mediate the beneficial effects on the body through hormetic mechanisms [16].

The antioxidant effect of H_2_ is mainly reflected in several aspects. H_2_ was first found to directly eliminate hydroxyl radicals and peroxynitrite. Compared with traditional antioxidants, such as superoxide dismutase (SOD), catalase, and *α*-tocopherol, H_2_ can easily penetrate biofilms and does not affect the normal metabolic redox reaction due to its small molecular weight and antioxidative activity which selectively affects only the strongest oxidant [17]. H_2_ can enhance the expression of the heme oxygenase-1 (HO-1) antioxidant by activating nuclear factor erythroid 2-related factor 2 (Nrf-2), an upstream regulating molecule of HO-1. H_2_ can also downregulate ROS directly or as a regulator of a gas-mediated signal. Further, by upregulating the expression of SOD and glutathione (GSH), and downregulating the expression of NADPH oxidase (NOX2) [18], H_2_ can significantly reduce ROS. Another study [19] showed that H_2_ mainly blocks the phosphorylation of ASK1 and its downstream signal molecules p38 MAPK or c-Jun N-terminal kinase (JNK), but not the production of ROS by NADPH oxidase.

The effects on the free radical chain reaction of lipid peroxidation may be another important mechanism of hydrogen antioxidation. Since Otha et al. reported at the 5th Symposium of Medical Molecular Hydrogen at Nagoya, Japan, in 2015 that exposure to a low concentration of hydrogen causes abnormal oxidation of phospholipids [20], many studies have established that H_2_ can protect cells from lipid and fatty acid peroxidation [21, 22]. Additionally, H_2_ can also reduce the expression of myeloperoxidase (MPO) [23] or decrease mitochondrial oxidoreductase activity [21] and stabilize mitochondrial membrane potential [24], so as to improve the tissue damage caused by oxidative stress.

### 2.3. Regulation of Endoplasmic Reticulum Stress

The accumulation of unfolded protein in the endoplasmic reticulum (ER) caused by pathological stress can trigger ER stress. Zhao et al. [18] observed that hydrogen inhalation significantly reduced the ER stress-related protein and alleviated tissue damage in myocardial I/R injury and later found that a mixture of H_2_ and O_2_ can block endoplasmic reticulum stress via the PKR-like ER-localized eIF2*α* kinase-eukaryotic initiation factor 2 alpha-activating transcription factor 4 (PERK-eIF2*α*-ATF 4), inositol-requiring enzyme 1-X-box binding protein 1 (IRE 1-XBP1), and ATF 6 pathways. A study of the relationship between H_2_ and ER stress in rats with I/R injury found that H_2_ induced the decrease of GRP78 and TNF receptor-associated factor 2 (TRAF2) expression [25], suggesting that the protective effects of H_2_ on myocardial I/R injury are related to decreased ER stress.

### 2.4. Regulation of Mitochondria

The accumulation of ROS can trigger the release of calcium from the ER, which results in the depolarization of mitochondria and the loss of the mitochondrial membrane potential [26]. The negative regulation of ROS and the inhibition of programmed cell death by H_2_ help maintain the structure and function of mitochondria. It was reported [27] that hydrogen treatment can block the opening of mitochondrial permeability transition pores in neurons during neurodegenerative disease. However, whether H_2_ can indirectly block these pores by reducing the production of ROS or acts directly is unclear. Early studies [28] showed that HRS moderated mitochondrial structural damage and simultaneously reduced microRNA- (miR-) 210 in hypoxic-reperfusion nerve tissue. However, recent studies have shown that the increase of miR-210 in injured tissues may be a compensatory action to maintain cell function and reduce ROS production [29, 30]. Whether H_2_ can directly inhibit miR-210 or indirectly reduce it by alleviating inflammation also remains unclear.

Mitochondrial damage caused by oxidative stress is an important cause of many neurodegenerative diseases. Early clinical experiments on Parkinson's disease [31] showed that H_2_ can significantly improve neurodegenerative symptoms with a therapeutic effect comparable to that of nonergot dopamine therapy. This cannot be explained by the antioxidative effect of H_2_, and thus, it was suggested that H_2_ may target mitochondria to improve the energy metabolism of cells. The observation that H_2_ treatment significantly improved the level of SH-SY5Y ATP and *Δψ*m in neuroblastoma [32] is an indication that H_2_ treatment can elevate energy metabolism in mitochondria by activating oxidative phosphorylation.

The observations of the conserved structural features shared between hydrogenases and the energy-converting complex I prompted the suggestion that H_2_ might serve as both a reductant and oxidant [24]. The hypothetical function of H_2_ in rectifying mitochondrial electron flow can explain the scavenging effect on ROS and the ability to slightly improve oxidative stress.

### 2.5. The Effects of Hydrogen on the Immune System

The main effect of H_2_ on the immune system is to reduce the production of immune active substances. Evidence suggests that H_2_ relieves inflammation in some autoimmune disorders, including rheumatoid arthritis (RA) [33, 34], dermatomyositis [35], and psoriasis [36]. However, whether H_2_ directly influences immune cells or organs remains unclear. Recent studies have found that H_2_ can relieve the dysregulated Th1/Th2 balance and can influence the number of T-regulatory cells (Tregs). H_2_ was first reported to restore Treg loss in a rat model of chronic pancreatitis [15] and was later proven to increase CD4+CD25+Foxp3+Treg cells and significantly reduce nasal mucosa damage in animals with allergic rhinitis, which may be secondary to the restoration of Th1/Th2 balance [37]. Upregulation of Tregs has been reported in cerebral I/R models [28]. This may be caused by the upregulation of tumor necrosis factor-beta 1 (TNF-*β*1) and downregulation of miR-21 or miR-210. H_2_ can also activate peroxisome proliferator-activated receptor-gamma coactivator-1 alpha (PGC-1*α*) to influence some kinds of T cells [38]. The specific mechanisms underlying the effects of H_2_ on immune cells remain to be defined.

### 2.6. Effects of H_2_ on Cell Death

#### 2.6.1. Prevention of Apoptosis

Apoptosis is a form of programmed cell death characterized by cell shrinkage, apoptotic body formation, karyorrhexis, and chromatin condensation. Apoptosis can be induced by both intrinsic and extrinsic signals. H_2_ exhibits antiapoptotic effects by up- or downregulating apoptosis-related factors. H_2_ inhibits the expression of the proapoptotic factors B-cell lymphoma-2-associated X-protein (Bax), caspase-3, caspase-8, and caspase-12 and upregulates the antiapoptotic factors B-cell lymphoma-2 (Bcl-2) and B-cell lymphoma-extra-large (Bcl-xl) [6]. Additionally, Terasaki and colleagues reported that H_2_ can downregulate the gene expression of proapoptotic Bax and inhibit its translocation to mitochondria by an unknown mechanism [39]. H_2_ can also inhibit apoptosis by activating the phosphatidylinositol-3-kinase/protein kinase B (PI3K/AKT) and the Janus kinase 2/signal transducer and activator of transcription 3 (JAK2-STAT3) signaling pathways in rats with myocardial ischemia-reperfusion injury (MIRI) [40, 41], as well as downregulating the p38 MAPK signaling pathway in rat models with lipopolysaccharide- (LPS-) induced acute lung dysfunction [42] and cerebral ischemia-reperfusion injury (CIRI) [43]. Interestingly, Wang et al. recently discovered that H_2_ inhibited the growth, migration, and invasion of the A549 and H1975 lung cancer cell lines and promoted cell apoptosis, suggesting that H_2_ might play crucial roles in the treatment of lung cancer [44]. Li et al. also revealed the apoptosis-inducing effect of H_2_ on KYSE-70 human esophageal squamous cell carcinoma cells [45]. Thus, H_2_ may protect normal cells from damage and suppressing cancer cells.

#### 2.6.2. Autophagy

Although the activation of autophagy can maintain the energy balance of cells through the degradation of macromolecular substances, excessive autophagy or autophagy-related stress triggered by stress stimuli will aggravate the inflammatory damage in tissues and organs. H_2_ plays a dual role in the regulation of autophagy. Under the regulation of H_2_, autophagy can be activated when protein aggregation becomes toxic and blocked once excessive autophagy causes damage to tissues. Zhuang et al. [46] showed that H_2_ treatment downregulated the expression of phosphomammalian target of rapamycin (p-mTOR)/mTOR and p62 in LPS-treated neuroglial cells and increased the expression of phospho-AMP-activated protein kinase (p-AMPK)/AMPK, light chain 3 (LC3) II/LC3 I, triggering receptor expressed on myeloid cells 2 (TREM-2), and Beclin-1 to activate autophagy and attenuate neuroinflammation in sepsis. Guan et al. [47] revealed that H_2_ can ameliorate chronic intermittent hypoxia-induced kidney injury by decreasing ER stress and inducing autophagy by inactivating oxidative stress-dependent p38 and JNK MAPKs. H_2_ can also inhibit autophagy by downregulating NF-*κ*B, Beclin-1, and MAPK and upregulating the HO-1, mTOR, and LC3B signaling pathways. Zhang et al. [42] found that saturated hydrogen saline alleviated LPS-treated lung injury and significantly reduced the expression of autophagy-related proteins, including LC3 and Beclin-1 (*P* < 0.05), suggesting that hydrogen saline can protect lung tissue against excessive autophagy. Saturated hydrogen saline can prevent excessive autophagy by eliminating excessive free radicals, reducing the concentration of free radicals in lung tissue, and promoting the expression of mTOR. HO-1 can function as an endogenous cytoprotective protein to assist in the prevention of oxidative stress and excessive cell autophagy. H_2_ can increase the tissue expression of HO-1 by promoting the expression of nuclear erythroid 2-related factor 2 (Nrf2) [48].

Toll-like receptors (TLRs) could be a potential target for H_2_ in autophagy regulation. TLR4, a key factor in the recognition of viral and bacterial factors, can be activated by LPS to induce autophagy of macrophages [49]. The inhibition of an LPS-induced inflammatory response by H_2_ supports the speculation that TLR may be a potential pathway of hydrogen-induced autophagy.

#### 2.6.3. Pyroptosis

Pyroptosis is an inflammatory programmed cell death pathway that protects multicellular hosts from invasive pathogens, including microbial infections [50]. Human and mouse caspase-1, human caspase-4 and caspase-5, and mouse caspase-11 act as essential activators of pyroptosis. While pyroptosis is normally beneficial for the host, excessive pyroptosis can result in sepsis and septic shock. Although there is no experimental data to explain the relationship between H_2_ and the pyroptosis pathway, it is conceivable that the regulation of some inflammatory factors and nuclear factors by H_2_ will interfere with the triggering of pyroptosis, or at least reduce pyroptosis-related inflammation. In one study [51], inhalation of 2% H_2_ reduced the expression of caspase-1, a key factor for pyroptosis activation. Physical rupture of cells caused by pyroptosis leads to the release of the proinflammatory cytokines IL-1*β* and IL-18, while hydrogen pretreatment can significantly reduce the level of these cytokines [52]. H_2_ has also been shown to regulate the expression of Atg7, which inhibits pyroptosis [53]. It has been proposed that HMGB1 [54] and IFN-*γ* [50] are necessary for caspase-11-dependent pyroptosis activation. The negative effect of H_2_ on the expression of these two factors may protect cells from pyroptosis. H_2_ is able to block the expression of caspase-3 [55], which serves both as the activator of apoptosis, and also blocks pyroptosis by cleaving gasdermin D [56]. Bidirectional crosstalk exists between the caspase-3 produced in apoptosis and the caspase in pyroptosis. The mechanism of this crosstalk remains unclear.

Human immunodeficiency virus (HIV) can accelerate the depletion of CD4+ T cells via interferon-gamma inducible protein 16- (IFI16-) triggered pyroptosis [50]. Thus, the regulation of pyroptosis by H_2_ may be a potential mechanism to treat HIV affection.

#### 2.6.4. Ferroptosis

Ferroptosis is a form of regulated cell death proposed by Dixon et al. [57] in 2012. Ferroptosis is accompanied by a lethal iron-dependent accumulation of lipid hydroperoxides. Although laboratory verification has not been forthcoming, we can still speculate that hydrogen can interfere with ferroptosis to alleviate inflammatory necrosis of tissues and organs in the pathological state, given the great deal of overlap between hydrogen regulation and ferroptosis pathways. It has been well-established that hydrogen has a negative regulatory effect on ROS. We speculate that the most critical redox imbalance in the process of ferroptosis will be eliminated by hydrogen; thus, ferroptosis will be blocked. In addition, H_2_ is able to block MAPK pathways, which are to prevent the depletion of reducing substances caused by iron ions and ROS [58]. A recent study [59] showed that HMGB1, which can be downregulated by hydrogen [9], can act as a positive regulator of ferroptosis via the RAS-JNK/p38 pathway.

HO-1 activity can be increased by hydrogen. HO-1 is a potential source of intracellular iron, and a recent study [60] demonstrated that HO-1-deficient renal epithelial cells were more sensitive to ferroptosis, indicating that free iron produced by HO-1 does not promote ferroptosis itself, and that HO-1 has an antiferroptotic effect. However, the effects of hydrogen on ferroptosis may not always be inhibitory. For example, miR-9, an inflammatory miRNA that is downregulated by hydrogen, can reduce the occurrence of ferroptosis [16]. The mechanism underlying the action of hydrogen on ferroptosis is yet to be fully clarified.

Some of the anti-inflammatory and antioxidation mechanisms of hydrogen are similar to those of GPX4 [61]. Both molecules have negative effects on the formation of lipid peroxide and NF-*κ*B. The combination of hydrogen and GPX4 activator may provide a new solution for the treatment of inflammation and other lipid peroxidation-mediated diseases.

#### 2.6.5. Circadian Clock

The circadian clock refers to an endogenous oscillator that controls 24 h physiological, metabolic, and behavioral processes. This clock is particularly crucial in maintaining homeostasis [62]. Intestinal microbiota, which regularly produce hydrogen gas in the process of the energy-producing anaerobic fermentation [63], undergo diurnal oscillations in composition and function [64]. In humans, the amount of hydrogen produced varies depending on the individual and the time of the day. Wilking et al. suggested that the circadian regulation of protein expression plays an important role in the cellular response to oxidative stress; they concluded that levels of byproducts of oxidative stress, such as protein damage, or lipid peroxidation, also oscillate with circadian rhythmicity, indicating circadian oscillations of oxidative stress responses. Thus, this rhythmicity of antioxidant levels can be exploited for a more precise targeting of ROS to offer better protection for the cells [65]. As the antioxidant activity of H2 has been widely verified, we suggest that H2 may exert a negative regulatory effect on ROS by regulating circadian rhythm, but there is yet no evidence regarding how H2 is involved in the regulation of circadian rhythm.

## 3. Preventive and Therapeutic Applications of H_2_

H_2_ has preventive and therapeutic effects on different system diseases (Table 1).

### 3.1. Effects of Hydrogen on the Respiratory System

The seventh edition of *Chinese Clinical Guidance for COVID-19 Pneumonia Diagnosis and Treatment (7^th^ edition)* issued by China National Health Commission recommends the inhalation of O_2_ mixed with H_2_. The recommendation recognized the efficacy of hydrogen in the treatment of respiratory diseases. A recent review summarized several researches on SARS-CoV-2 and pointed out that the possibility that H_2_ could alleviated SARS-CoV-2 infection though affecting cellular responses [66]. Antiviral efficacy and safety of H2 in treating NCP patients have attracted the attention of researchers in completed or ongoing multicenter clinical trials. Guan et al. [67] evidenced that inhalation of H_2_/O_2_ mixed gas efficiently improved respiratory symptoms especially dyspnea as well as disease severity without observed side effects. Another multi-centre RTC (ChiCTR2000030258) aims verifying the efficacy and safety of H_2_/O_2_ mixed gas is ongoing. Due to its small molecular weight, hydrogen in the inhaled gas mixture can reduce airway resistance, increase oxygen dispersion, and increase oxygen flow. COVID-19 can provoke an inflammatory storm by excessively activating the immune system, causing severe inflammatory damage to the lungs and extrapulmonary tissue, which is also the main cause of death [68].

A study involving 41 patients with NCP showed that patients in the intensive care unit displayed a significantly higher level of inflammatory factors that included IL-2, IL-7, IL-10, and TNF-*α* and that most of these factors could be downregulated by hydrogen [69]. We speculate that the application of hydrogen may reduce the risk of inflammatory storm and thus prevent severe effects. Many NCP patients need ventilator-assisted therapy. Inflammatory changes in the respiratory system make lung tissue prone to ventilator-induced lung injury (VILI), even with low tidal volume [70]. Ventilation with 2% H_2_ was proved to be able to downregulate the mRNAs for proinflammatory mediators such as TNF-*α*, IL-1*β*, Egr-1, and CCL2 and induced antiapoptotic genes including Bcl-2 and Bcl-xL. It is consistent with the histopathological results which showed that inflammatory cell infiltration and bronchial epithelial apoptosis were improved in VILI mice after H_2_ treatment. H_2_ has a potential to protect human lung tissues from VILI as well via its anti-inflammatory, antioxidant, and antiapoptotic effects [11]. Moreover, hydrogen can also increase surfactant proteins to prevent further lung injury [11]. One study reported that chest computed tomography scans performed at the early stage in patients that ultimately developed in severe infection revealed multiple small flake shadows and interstitial changes, suggesting that pulmonary fibrosis affects the prognosis of the disease. HRS has been reported to reverse epithelial-mesenchymal transition (EMT) and prevent pulmonary fibrosis by inhibiting oxidative stress and increasing the expression of E-cadherin [71].

Hydrogen also has the potential to protect lung tissues from pulmonary hypertension [72], sepsis [73], smoke inhalation injury [74], hemorrhagic shock and resuscitation [75], and other toxic substances/events. In animal models, hydrogen also affects asthma and chronic obstructive pulmonary disease [76].

### 3.2. Effects of Hydrogen on the Nervous System

Myriad forms of irreversible damage that occur in nervous system diseases are often caused by neuroinflammation, excessive oxidative stress, mitochondrial dysfunction, and cell death. The therapeutic effects of hydrogen on the nervous system have been verified in animal models and clinical trials. Hydrogen can reportedly reduce the loss of dopaminergic neurons and can improve nigrostriatal degeneration a mouse model of Parkinson's disease (PD) following treatment with 6-hydroxydopamine [77] and 1-methyl-4-phenyl-1,2,3,6-tetrahydropyridine [78]. A recent clinical trial showed that HW can improve the total Unified Parkinson's Disease Rating Scale score of PD [31]. Early clinical trials revealed that hydrogen improves PD through antioxidation. More recent research found that hydrogen may slightly increase oxidative stress or act as the rectifier of the mitochondrial electron flow and improve PD by regulating mitochondrial energy [24] or via hormetic mechanisms. By hormetic mechanisms, H_2_ will simulate strenuous exercise to generate a mild increase of ROS which will evoke hormesis and then activate the Nrf2, NF-*κ*B pathways, and heat shock responses to protect neurons and other tissues [16]. Hydrogen also improved cognitive function in Alzheimer's patients in a clinical trial [79]. The collective findings indicate the safety and effectiveness of hydrogen in the treatment of neurodegenerative diseases.

Hydrogen has also been shown to protect the nervous system of a fetus or newborn. In two case studies, pregnant women who had become infected with COVID-19 displayed a high delivery rate of fetuses with intrauterine distress leading to a higher incidence of perinatal hypoxic-ischemic brain damage [77, 78]. The findings indicate the necessity of measures to prevent neonatal encephalopathy or reduce neonatal neurological deficit for pregnant women infected with COVID-19. H_2_ can inhibit the activation of proinflammatory cytokines, microglia [79], and 8-hydroxy dehydrogenase (8-OHdG) [80] to reduce oxidative damage and neuroinflammation in the fetal brain in animal models. The protective effects have been verified by clinical trials. Hippocampal damage caused by I/R injury in the uterus on day 7 after birth was reportedly improved for pregnant women who had been treated with HW and was associated with reductions of 4-hydroxy ketone and 8-OHdG [81]. The available data support the possibility that hydrogen therapy could protect fetuses of pregnant women infected with COVID-19. In addition, hydrogen can also protect against spinal cord injury and a variety of brain injuries caused by ischemia, hypoxia, trauma, and hemorrhage. A clinical study of patients suffering from cerebral infarction reported that hydrogen inhalation improved imaging results, National Institutes of Health Stroke Scale scores used to quantify the severity of stroke, and physical therapy evaluations based on the Barthel Index [82]. A rat model of subarachnoid hemorrhage revealed the influence of hydrogen in lessening neurological deficits [83] and endothelial cell injury [84]. In rats, hydrogen can also relieve neuropathic pain [85] after spinal cord injury and can ameliorate neurotoxicity [69].

### 3.3. Effects of Hydrogen on Cardiovascular Diseases

With the acceleration of urbanization and aging of global societies, the risk of cardiovascular diseases (CVD) has increased. The World Health Organization ranks CVD as the leading cause of death globally, accounting for 17.5 million deaths annually. Two of every five deaths in China are attributed to CVD, which exceeds the death rate due to cancer or other diseases [86]. However, most clinical trials to date have failed to effectively prevent and treat CVD. Thus, novel therapies are urgently required.

During the past decade, many basic and clinical studies have provided evidence supporting the view that H_2_ treatment protects against CVD and improves cardiac function. Ohsawa et al. discovered that consumption of HW for 6 months reduced the oxidative stress level and the volume of atherosclerotic lesions derived from macrophage accumulation in apolipoprotein E knockout mice (ApoE-/- mice) [87]. Iketani et al. recently revealed that continuous administration of HW in low-density lipoprotein (LDL) receptor-deficient mice decreased the numbers of endothelial cells in the atheroma expressing the senescence factors p16INK4a and p21, as well as suppressing macrophage infiltration and TNF-*α* expression in the atheroma, suggesting that vascular aging can be suppressed by HW [88]. Another study demonstrated increased flow-mediated dilation in the high-H_2_ group in which eight males and females drank HW containing 7 ppm H_2_, indicating that H_2_ may protect the vasculature from shear stress-derived ROS that is detrimental [89].

In addition to treating atherosclerosis, H_2_ reduces MIRI, which refers to a heart lesion that develops upon the resumption of the flow of oxygen-rich blood after a period of ischemia and which usually occurs during acute myocardial infarction or open-heart surgery [40, 90]. A recent series of studies by Li et al. found that HW inhibited cardiomyocyte apoptosis by activating the PI3K/AKT and JAK2-STAT3 signaling pathways and can also reduce the level of oxidative stress in myocardial tissue by upregulating the expression of the nuclear erythroid 2-related factor 2/antioxidant response element (Nrf2/ARE) signaling pathway, which alleviated I/R injury in isolated rat hearts [39, 40, 91]. Other studies demonstrated that intraperitoneal injection of HW before reperfusion significantly decreased the concentration of malondialdehyde (MDA) and infarct size, as well as reducing myocardial 8-OHdG and the levels of TNF-*α* and IL-1*β* in an area at risk zones [92, 93]. Moreover, Qian et al. described the hydrogen-mediated protection of myocardium degeneration due to radiation-induced injury in rats [94]. Treatment with HRS was shown to ameliorate vascular dysfunction, including aortic hypertrophy and endothelial function, in spontaneously hypertensive rats by abating oxidative stress, restoring baroreflex function, preserving mitochondrial function, and enhancing nitric oxide bioavailability [95]. In another study, the intraperitoneal administration of HRS improved hemodynamics and reversed right ventricular hypertrophy in male Sprague-Dawley rats with pulmonary hypertension induced by monocrotaline [96]. H_2_ inhalation is also a favorable strategy to mitigate mortality and functional outcome of postcardiac arrest syndrome in a rat model [97]. These collective findings indicate the potential of H_2_ in novel therapeutic approaches for the clinical treatment of CVD.

### 3.4. Effects of Hydrogen on Digestive System Diseases

Hepatic ischemia-reperfusion injury (HIRI) is common in liver surgery and liver transplantation. H_2_ treatment ameliorated HIRI in a mouse fatty liver model by reducing hepatocyte apoptosis, inhibiting macrophage activation and inflammatory cytokines, and inducing HO-1 and Sirt1 expression [98]. Inhalation of high concentrations of hydrogen significantly improved liver function in a mouse HIRI model by activating the A2A receptor-mediated PI3K-Akt pathway [99]. A recent series of studies also discovered that portal vein injection of HRS in miniature pigs with laparoscopic HIRI promoted liver function recovery and liver regeneration by reducing hepatocyte autophagy and apoptosis and inhibited ER stress, with significant hepatoprotective effects observed [100–103]. Hydrogen flush after cold storage refers to an end-ischemic H_2_ flush directly to donor organs ex vivo. This approach can significantly protect liver grafts from IRI [104], providing a potentially effective strategy for organ preservation. Other studies demonstrated the protective effects of H_2_ in liver damage induced by parasites [105], obstructive jaundice [106], shock and resuscitation [107], sepsis [108], doxorubicin [6], and aflatoxin B1 [109]. In a mouse model of nonalcoholic fatty liver disease (NAFLD), HW downregulated Nrf2-mediated miR-136 expression by targeting the maternally expressed 3 long noncoding RNA gene [110], providing a rationale for further clinical trials. In a human study, HW also significantly reduced liver fat accumulation in twelve overweight outpatients with NAFLD [111]. Another in vivo study revealed that oral HW significantly attenuated oxidative stress in patients with chronic hepatitis B [112]. In recent years, it has become widely accepted that bile acids are a nutrient signaling hormone [113]. Molecular hydrogen was recently demonstrated to participate in the regulation of bile acid metabolism, particularly in the inhibition of bile acid oxidation, in some gut bacteria [114].

Intestinal I/R injury is a multifactorial pathophysiological process with high morbidity and mortality. I/R injury occurs in a variety of clinical settings that include major cardiovascular surgery, trauma, shock, and small intestinal transplantation [115]. Yao et al. recently observed that intraperitoneal injection of H_2_ attenuated I/R-induced mucosal injury and apoptosis of epithelial cells in mice by regulating miRNAs, in particular by regulating miR-199a-3p [116]. Furthermore, HW reportedly inhibited intestinal I/R-induced oxidative stress, apoptosis, and inflammation and promoted epithelial cell proliferation in rats, which protected against intestinal contractile dysfunction and damage induced by intestinal I/R [117–119]. Most gastrointestinal microbial species encode the genetic capacity to metabolize H_2_, meaning that H_2_ might affect the gut bacterial composition [120], and bacterial translocation is an important cause of multiple organ dysfunction syndromes in critical illness. Ikeda et al. described that the luminal administration of HW prevented intestinal dysbiosis, hyperpermeability, and bacterial translocation in a murine model of sepsis [121]. In another study, the inhalation of 2% H_2_ also attenuated intestinal injuries caused by severe sepsis in male Nrf2 KO mice by regulating HO-1 and HMGB1 release [122]. Moreover, an in vivo study revealed that H_2_ inhalation improved the prognosis in patients with stage IV colorectal cancer by activating PGC-1*α* and restoring exhausted CD8+ T cells [123].

Wang et al. interestingly discovered that cytotoxin-associated gene A cytotoxin, a virulence factor of *Helicobacter pylori* that augments the risk of gastric cancer, can be delivered into host cells by the H_2_-utilizing respiratory chain of the bacterium, extending the roles of H_2_ oxidation to include transport of a carcinogenic toxin [124]. Although this study indicated that H_2_ may play a role in increasing gastric cancer risk, abundant studies also demonstrated that H_2_ is protective in gastric damage induced by oxidative stress [125] and aspirin [126]. Franceschelli et al. found that electrolyzed reduced water, which is rich in molecular hydrogen, rapidly improved symptoms in patients with gastroesophageal reflux disease [127]. H_2_ treatment also controlled the severity of chronic pancreatitis [15] and acute necrotizing pancreatitis [128].

### 3.5. Effects of Hydrogen on Reproductive System Diseases

Yang et al. recently demonstrated using a mouse model of human endometrial tumor xenograft that HW has an antitumor effect that was sufficient to inhibit xenograft volume and weight of endometrial tumors via the ROS/NLRP3/caspase-1/GSDMD-mediated pyroptotic pathway, indicating a biphasic effect of H_2_ on cancer that involves the promotion of tumor cell death and protection of normal cells [129]. Other authors reported that H_2_ inhalation reduced the size of the endometrial explants, inhibited cell proliferation, improved SOD, and regulated the expression of matrix metalloproteinase 9 and cyclooxygenase 2 in an endometriosis rat model [130]. HRS is effective in attenuating ovary injury induced by I/R [131] and cisplatin [132]. HW improved serum anti-Müllerian hormone levels and reduced ovarian granulosa cell apoptosis in a mouse immune premature ovarian failure model induced by zona pellucida glycoprotein 3 [133]. In a hemisectioned spinal cord injury rat model, HRS inhibited the injury-induced ultrastructural changes in gonadotrophs, ameliorated the abnormal regulation of the hypothalamic-pituitary-testis axis, and thereby promoted the recovery of testicular injury [134]. In irradiated rats, HRS improved testis weight, testis dimensions, sperm count, sperm motility, and serum testosterone levels [135]. HW stimulated spermatogenesis as well as increased sperm production and sperm motility in mice of different ages [136]. Based on previous studies, Begum et al. hypothesized that H_2_ may modulate intracellular MAPK cAMP and Ca^2+^ signals involved in testosterone hormone production to improve male fertility caused by redox imbalance [137]. Finally, H_2_ decreased the percentage of sperm abnormalities and improved sperm morphology following the prolonged exposure of mouse low doses of radiation [138]. The collective data indicate that hydrogen can protect both female and male fertility.

### 3.6. Effects of Hydrogen on Urinary System Diseases

Acute kidney injury (AKI) is an important risk factor for the development of chronic kidney disease. Wu et al. recently found that saturated hydrogen alleviates CCL4-induced AKI via JAK2/STAT3/p65 signaling [139]. Inhalation of a hydrogen-rich aerosol appears to be very useful for renal protection and inflammation reduction in septic AKI, based on the observations of increased anti-inflammatory cytokine (IL-4 and IL-13) mRNA levels in renal tissues and increased macrophage polarization to the M2 type, which generates additional anti-inflammatory cytokines (IL-10 and transforming growth factor-beta, TGF-*β*) [140]. Additionally, H_2_ can alleviate AKI induced by I/R [141], liver transplantation [142], burns [143], and sodium taurocholate-induced acute pancreatitis [144]. Recently, Lu et al. demonstrated that HW can restore a balanced redox status and alleviate cyclosporine A-induced nephrotoxicity by activating the Keap1/Nrf2 signaling pathway [145]. H_2_ can ameliorate kidney injury induced by chronic intermittent hypoxia by decreasing ER stress and activating autophagy through the inhibition of oxidative stress-dependent p38 and JNK MAPK activation [46]. HW also reportedly can inhibit the development of renal fibrosis and prevent HK-2 cells from undergoing EMT mediated through Sirt1, a downstream molecule of TGF-*β*1. HRS markedly reduced interstitial congestion, edema, and hemorrhage in renal tissue, prevented renal injury, and promoted renal function recovery after I/R injury in rats through antiapoptotic and anti-inflammatory actions in kidney cells [146]. Other authors described that HW significantly reduced the increased postvoid residual volume in obstructed rats and ameliorated bladder dysfunction secondary to bladder outlet obstruction by attenuating oxidative stress [147].

### 3.7. Effects of Hydrogen on Metabolic Syndrome

Metabolic syndrome is associated with excess calorie intake and encompasses a range of medical conditions that include obesity, insulin resistance, and dyslipidemia. Many studies have demonstrated the protective effects of H_2_ in metabolic syndrome. Qiu et al. reported that saturated hydrogen decreased total cholesterol, total glyceride, and LDL, increased high-density lipoprotein in the peripheral blood, and alleviated the activity of isocitrate lyase, suggesting that H_2_ could improve lipid metabolism disorders by inhibiting the glyoxylic acid cycle [148]. Glucose and insulin levels in the serum were also significantly lower in H_2_-treated mice, which markedly improved type 2 diabetes mellitus and diabetic nephropathy-related outcomes [149]. Moreover, gut-derived hydrogen production induced by L-arabinose reportedly had beneficial effects on metabolic syndrome in C57BL/6J mice fed a high-fat diet [150] and reduced oxidative stress and the peripheral blood IL-1*β* mRNA level in sixteen type 2 diabetic patients [151]. H_2_ treatment has also shown positive effects on energy metabolism. In 2011, Kamimura et al. reported that prolonged consumption of HW significantly controlled fat and body weights in db/db obese mice by stimulating energy metabolism [152]. A recent study revealed that H_2_ attenuated allergic inflammation in a mouse model of allergic airway inflammation by reversing an energy metabolic pathway switch from oxidative phosphorylation to aerobic glycolysis [153].

### 3.8. Effects of Hydrogen on Motor System Diseases

Although many studies have investigated the effectiveness of H_2_ on various diseases related to oxidative stress, little is known about the influence of H_2_ on exercise-induced oxidative stress. In 2012, Aoki et al. found that HW reduced blood lactate levels and improved exercise-induced decline of muscle function in ten male soccer players [47]. Yamazaki et al. discovered that the serum 8-OHdG levels in H_2_-treated race horses were significantly suppressed, strongly suggesting a protective effect of H_2_ in exercise-induced, ROS-mediated detrimental tissue damage [154]. Additionally, hydrogen bathing attenuated exercise-induced muscle damage and delayed-onset muscle soreness but had no effects on the peripheral neutrophil count and both dynamics and functions of neutrophils [155]. These findings highlight that further studies are needed to clarify the mechanisms of H_2_. Inhalation of H_2_ significantly decreased infarct zone and area with loss of tissue structure, attenuated muscle damage, and enhanced functional recovery in a mouse hindlimb I/R injury model [156]. Finally, Hasegawa et al. revealed that H_2_ improved muscular dystrophy in the mdx mouse model for Duchenne muscular dystrophy [157].

### 3.9. Effects of Hydrogen on Sensory System Diseases

Hydrogen has a therapeutic role in alleviating the damage to some sensory organs, mainly through antioxidation. Hydrogen promotes wound healing in tissue or protective barriers including skin and mucosa. For example, preinhalation of hydrogen-containing gas decreased wound healing time in a rat model of radiation-induced skin injury [158]. Other authors reported that H_2_ inhalation reduced the wound area and levels of proinflammatory cytokines in pressure ulcers [159]. Moreover, hydrogen can improve skin lesions in some immune disorders by interfering with the immune system or ROS removal [160]. HW also benefits the wound healing process of the oral palate. Hydrogen also may protect hearing and vision. Kurioka et al. [161] demonstrated that hydrogen inhalation significantly reduced outer hair cell loss and improved auditory brainstem response after noise exposure, indicating a protective effect for noise-induced hearing loss. Hydrogen has been shown to be effective in treating cornea injury caused by alkali [162], fluoride, chloropicrin [163], and ultraviolet B radiation [164].

### 3.10. Effects of Hydrogen on Cancer

Many animal models have established the efficacy of hydrogen against cancers. The attributes of hydrogen include blocking of the regulator for chromosome condensation [43], some crucial molecules in stemness [165], proliferation [123], and angiogenesis [165], and the alleviation of oxidative stress. The combination therapy of hydrogen and other novel antineoplastic drugs, such as LY294002 [166], which is an inhibitor of PI3K, has demonstrated great potential and efficacy. An increasing number of clinical trials are being carried out. A recent survey [140] on 82 advanced cancer patients exemplified that hydrogen can control cancer progression and improve the quality-of-life. Akagi [37] treated 55 stage IV colorectal carcinoma patients using hydrogen inhalation and documented enhanced mitochondrial activity due to the activation of PGC-1*α* to reduce the proportion of terminal PD-1+ CD8+ T cells. The depletion of these cells is associated with improved cancer prognosis [37, 123]. This therapeutic effect has also been confirmed in another trial conducted in one patient with metastatic gallbladder cancer [167]. In a case report in 2019, hydrogen gas therapy resulted in 1the disappearance of the metastatic brain tumors in a woman diagnosed with lung cancer [168]. Finally, hydrogen can also reduce the side effects of cisplatin [169] and radiotherapy [170]. Though growing evidences have shown the effects of H2 on alleviating both cancer progression and side effects of chemotherapeutics, the H2 therapy applied in cancer is just in a nascent stage. At present, the published researches on the anticancer effects of H2 mainly focus on lung cancer [168], colorectal cancer [37], and glioma [165]. It remains unclear how many cancers can effectively be alleviated by H_2_ and how many can not be.

At present, patients with COVID-19 pneumonia are usually treated with high flow pure oxygen (without adding H_2_), although the effect of O_2_ when associated with H_2_ may give better results [171]. The production of mucus in these patients reduces the absorption of O_2_, while with a mixture of O_2_ and H_2_, the bronchioles and the alveoli of lungs are further expanded, optimizing the absorption of O_2_ [172]. H_2_ is used as a catalyst to accelerate the binding of hemoglobin with O_2_ and the release of hemoglobin with carbon dioxide [173].

## 4. Conclusions

Hydrogen has great potential in the regulation of oxidative stress, inflammation, energy metabolism of organelles, and programmed cell death. Many animal experiments and clinical trials have established the protective effects of hydrogen on many organs and systems.

Research in this area has increased over the past 15 years. However, the details of the specific molecular mechanisms of the therapeutic effects of hydrogen remain unclear. For example, whether hydrogen can truly be used to regulate ferroptosis, pyroptosis, or the circadian clock is not known. Since H_2_ is not something like rapamycin or leucine only going to have one direction (opposite) effects on autophagy, is it possible to regulate autophagy or apoptosis in a specific direction? Previous studies have clearly explained the antioxidative stress effect of hydrogen. However, some recent clinical trials have shown that H_2_ can induce oxidative stress in some cases as well. Ventilation with H_2_ can induce a mild increase of ROS to activate the Nrf2, NF-*κ*B pathways, and heat shock responses. H_2_-induced ROS production can also be observed in cancer cells. The specific mechanism underlying the hydrogen-induced increase of oxidative stress should be explained by more experiments. These and other questions concerning the mechanism of hydrogen should be further explored.

There are many factors that limit the clinical use of hydrogen. Firstly, hydrogen is considered unsafe at concentrations above 4% because such a high level of H_2_ is explosive and might bring cytotoxic effects. Previous studies have indicated that the concentration of hydrogen should be stabilized beyond 2% to enable protection from acute oxidative stress. However, even 2% of hydrogen is not absolutely safe. Most clinical ventilators are equipped with platinum hot manometers, because H_2_ and O_2_ can overheat the platinum surface at room temperature. Secondly, there is a lack of specialized devices that enable the administration of effective hydrogen concentrations, while ensuring that they are safe. Thirdly, there have been few large-scale controlled human studies on the effects of hydrogen. Fourthly, Liu and his colleagues demonstrated that the inhalation of H_2_ resulted in a slower elevation of the H_2_ concentration than that achieved with intraperitoneal, intravenous, or oral administration. However, the elevated H_2_ concentrations were maintained for at least 60 minutes after inhalation. Thus, it should be deliberated to choose the administration of H_2_ [7]. As a result, the dose-specific effects or side effects of hydrogen in humans remain unclear.

The data regarding the known mechanisms underlying the action of hydrogen indicate that hydrogen can alleviate the damage in multiple organs in NCP patients. The comparisons of the different modalities of hydrogen indicate the value of HW in the effective treatment of such patients.

Hydrogen is inexpensive and safe and can be administered through many ways. We anticipate that as large-scale clinical trials confirm the therapeutic efficacy and safety of hydrogen, its full clinical potential will be realized.

## Figures and Tables

**Table 1:** Mechanisms of H_2_ in multiple systemic diseases.

Diseases	Effects of H_2_	Reference
Respiratory system	Regulates IL-2, IL-7, IL-10, and TNF-*α*	[66]
	Increases surfactant proteins	[11]
	Reverses EMT and increases E-cadherin	[68]
Cardiovascular system	Suppresses macrophage infiltration, TNF-*α* expression, and vascular aging	[88]
	Inhibits cardiomyocyte apoptosis	[39, 40]
	Decreases MDA, 8-OHdG, and IL-1*β*	[92, 93]
Nervous system	Reduces loss of dopaminergic neurons and improves nigrostriatal degeneration	[74, 75]
	Reduces neurological deficits and endothelial cell injury	[83, 84]
Digestive system	Induces HO-1 and Sirt1 expression	[98]
	Activates the A2A receptor-mediated PI3K-Akt pathway	[99]
	Inhibits bile acid oxidation	[114]
Reproductive system	Inhibits cell proliferation and improves SOD	[130]
	Improves serum levels of anti-Müllerian hormone	[133]
	Improves serum testosterone level	[135]
Urinary system	Increases Il-4 and Il-13 and promotes macrophage polarization to the M2 type	[140]
	Activates the Keap1/Nrf2 signaling pathway	[145]
	Prevents HK-2 cells from undergoing EMT	
Motor system	Reduces blood lactate levels	[47]
	Suppresses serum 8-OHdG levels	[154]
Sensory system	Reduces wound area and levels of proinflammatory cytokines	[159]
	Improves auditory brainstem response	[161]
Metabolic syndrome	Decreases LDL and increases high-density lipoprotein	[148]
	Decreases glucose and insulin levels	[149]
	Stimulates energy metabolism	[152]
Cancer	Controls cancer progression and improves quality-of-life	[140]
	Reduces proportion of terminal PD-1+ CD8+ T cells	[37]

## Abbreviation

NCP: Novel coronavirus pneumonia
H_2_: Hydrogen
I/R: Ischemia/reperfusion
HRS: H_2_-rich saline
HW: H_2_-rich water
ROS: Reactive oxygen species
ER: Endoplasmic reticulum
EMT: Epithelial-mesenchymal transition
CVD: Cardiovascular diseases
NAFLD: Nonalcoholic fatty liver disease
AKI: Acute kidney injury
MDA: Malondialdehyde
Nrf2: Nuclear erythroid 2-related factor 2
PGC-1*α*: Peroxisome proliferator-activated receptor-gamma coactivator-1 alpha.
